# Plasmodium falciparum YTH2 Domain Binds to m6A-Containing mRNA and Regulates Translation

**DOI:** 10.1128/mBio.01367-21

**Published:** 2021-11-23

**Authors:** Gayathri Govindaraju, Sreenivas Chavali, Arumugam Rajavelu

**Affiliations:** a Pathogen Biology, Rajiv Gandhi Centre for Biotechnology (RGCB), Thiruvananthapuram, Kerala, India; b Department of Biology, Indian Institute of Science Education and Research (IISER) Tirupati, Tirupati, Andhra Pradesh, India; c Department of Biotechnology, Bhupat & Jyoti Mehta School of Biosciences, Indian Institute of Technology Madras, Chennai, Tamil Nadu, India; University of Georgia

**Keywords:** Epigenetics, *Plasmodium falciparum*, malaria, methylation

## LETTER

**FIG 1:**
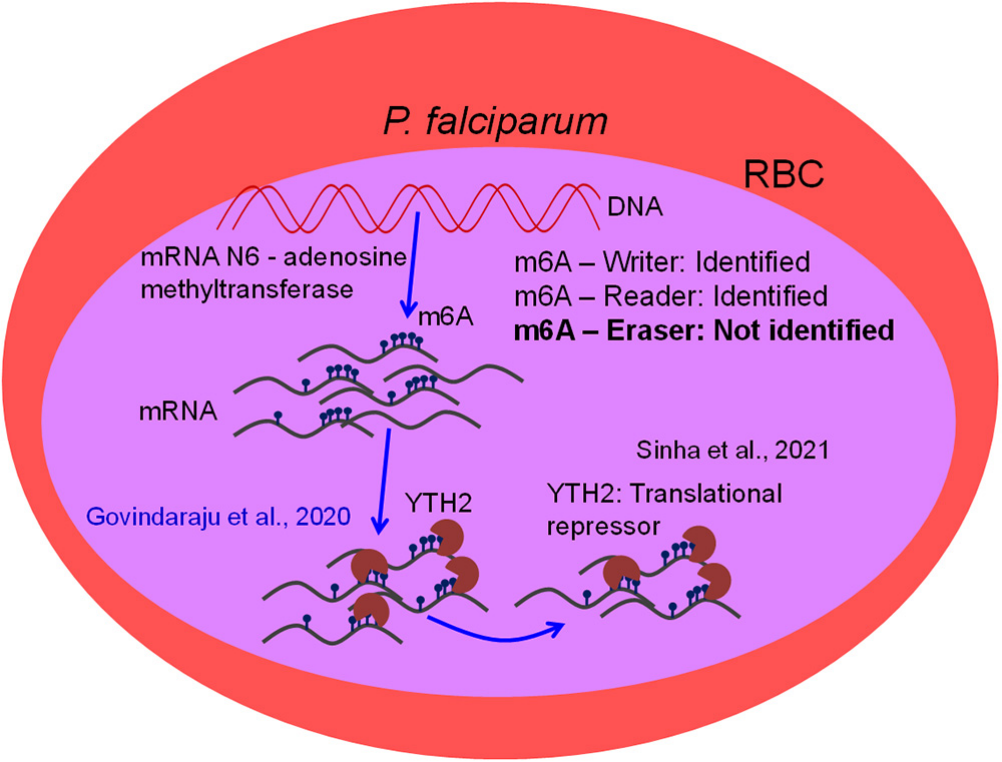
Schematic representation of PfYTH2 functions in human malaria parasite P. falciparum. The PfYTH2 protein was characterized and reported by Govindaraju et al. (5) in 2020, and subsequently, Sinha et al. (6) in 2021 showed that PfYTH2 functions as a translational repressor in P. falciparum.

Plasmodium falciparum is an important pathogen that causes severe malaria in humans. The malaria parasite carries unique epigenetic signatures, which are required for optimal gene expression in the parasite during its development in red blood cells (RBCs) (1–3). A recent study showed that P. falciparum mRNA contains a significant amount of epitranscriptomic modifications (N^6^-methyladenosine [m6A]) and that its presence on various transcripts in the parasite is highly dynamic (4). Subsequently, we reported an extensive characterization of the P. falciparum YTH2 (PfYTH2) protein that specifically interacts with m6A-containing mRNA of P. falciparum (5). The PfYTH2 protein has the conserved methyl-binding pocket which is formed by aromatic amino acids, and we found that F98 amino acid is essential in mediating the interaction of YTH2 to m6A-containing mRNA of P. falciparum (5). Importantly, in a recently published article in *mBio*, Sinha et al. (6) reported similar findings that PfYTH2 binds to m6A-containing mRNA through additional experiments. Beyond this, Sinha et al. showed that PfYTH2 protein binds to translation machinery and functions as a translational repressor in P. falciparum (6). Specifically, two major findings of Sinha et al. (6) overlap with our study published in August 2020 (5). The first finding was the m6A-specific interaction of PfYTH2 protein. We used a modified methylated RNA immunoprecipitation (MeRIP) assay, followed by dot blot assay to establish this, while Sinha et al. used oligonucleotide pulldown assay and MeRIP assay, followed by a dot blot assay by to report the same. Second, we had reported the binding strength of PfYTH2 to m6A-containing RNA oligonucleotides by performing molecular dynamics (MD) simulations, site-directed mutagenesis, followed by MeRIP assay, and fluorescence depolarization assay. We reported that W46 amino acid is the part of the methyl-binding pocket in PfYTH2 protein, and Sinha et al. have also identified W46 as an important residue, by using site-directed mutagenesis, followed by dot blot assay. With such overlap of findings, we are surprised to note that our work has not been cited in the recent article published by Sinha et al. (6). We see the overlapping observations as an independent validation of our findings by Sinha et al. We reiterate that we reported the identification and functional/biochemical characterization of PfYTH2 protein, and in the subsequent study, Sinha et al. showed the translational repressor functions of PfYTH2 protein in P. falciparum (Fig. 1). The apicomplexan parasites exhibit dynamic translational plasticity during its various developmental stages and the discovery of epitranscriptomic modification and its reader domain protein in P. falciparum open new avenues in understanding the translational plasticity of these parasites (4–6). We strongly believe that it is paramount to highlight the time line of discoveries of epitranscriptomic modifications and its reader domain protein in human malarial parasite and give credit where it is due, as part of best practice of science publishing. We envisage that the foundation laid by these discoveries will motivate many upcoming research studies on the role of epitranscriptome machinery in the translational plasticity of the apicomplexan parasites and in developing intervention strategies to tackle malaria using this knowledge.

## References

[1] Merrick CJ, Duraisingh MT. 2010. Epigenetics in Plasmodium: what do we really know? Eukaryot Cell 9:1150–1158. doi:10.1128/EC.00093-10. 20562224 PMC2918939

[2] Cui L, Miao J. 2010. Chromatin-mediated epigenetic regulation in the malaria parasite Plasmodium falciparum. Eukaryot Cell 9:1138–1149. doi:10.1128/EC.00036-10. 20453074 PMC2918932

[3] Voss TS, Bozdech Z, Bártfai R. 2014. Epigenetic memory takes center stage in the survival strategy of malaria parasites. Curr Opin Microbiol 20:88–95. doi:10.1016/j.mib.2014.05.007. 24945736

[4] Baumgarten S, Bryant JM, Sinha A, Reyser T, Preiser PR, Dedon PC, Scherf A. 2019. Transcriptome-wide dynamics of extensive m(6)A mRNA methylation during Plasmodium falciparum blood-stage development. Nat Microbiol 4:2246–2259. doi:10.1038/s41564-019-0521-7. 31384004 PMC7611496

[5] Govindaraju G, Kadumuri RV, Sethumadhavan DV, Jabeena CA, Chavali S, Rajavelu AN. 2020. N6-adenosine methylation on mRNA is recognized by YTH2 domain protein of human malaria parasite Plasmodium falciparum. Epigenetics Chromatin 13:33. doi:10.1186/s13072-020-00355-7. 32867812 PMC7457798

[6] Sinha A, Baumgarten S, Distiller A, McHugh E, Chen P, Singh M, Bryant JM, Liang J, Cecere G, Dedon PC, Preiser PR, Ralph SA, Scherf A. 2021. Functional characterization of the m6A-dependent translational modulator PfYTH.2 in the human malaria parasite. mBio 12:e00661-21. doi:10.1128/mBio.00661-21. 33906926 PMC8092261

